# New Drugs and Preparations

**Published:** 1894-10-13

**Authors:** 


### NEW DRUGS AND PREPARATIONS.

[We shall be glad to receive, at our Office, 428, Strand, London, W.C., from the manufacturers, specimens of all new preparations and drugs which may be brought out from time to time.]

### TOLYSAL.

This body is the salicylate of tolypyrine. It is crystalline, slightly pink in colour, bitter and astringent in taste, slightly soluble in water, freely in alcohol. Its action has been studied by Hennig (Deutsch med. Wochenschr., 1893, 193), who found in rabbits and guinea-pigs that doses of 45 grains per day did not produce poisonous symptoms. In various febrile and rheumatic affections he summarises its action as follows: 1. In daily divided doses of 45 to 90 grains, tolysal may be considered a certain remedy in acute articular rheumatism. 2. Given in the same dose for several days it was efficacious in chronic articular and muscular rheumatism. After the pain has subsided it is necessary to continue its administration in smaller doses for some days to prevent relapse. 3. In 15 to 45 grain doses it has energetic antineuralgic effects, which are often lasting. 4. It is an active antipyretic; in 60 to 120 grains daily it lowers the temperature satisfactorily, the pulse and respiration falling at the same time. The fall begins in about an hour after administration, and the more slowly it occurs the more prolonged is its duration. 5. It lowers normal temperature only to a very limited degree. 6. In rheumatism it sometimes succeeds after other usual remedies have failed to influence the course of the disease. 7. It has no cumulative action, nor is tolerance to its antipyretic effects induced. 8. As an antipyretic it is most efficacious when administered in the afternoon, and small doses frequently repeated are less active than larger doses repeated at longer intervals. 9. It acts to some extent as an hypnotic. 10. No poisonous or disagreeable after effects were ever noticed, and in this respect it is superior to quinine, antipyrine salicylate of sodium, and most other antipyretics. 11. It is antiseptic. In conclusion, the author recommends it very strongly as an antipyretic, antineuralgic, and antirheumatic. It is relatively cheap and seems to be quite free from ill effects.

### XANTHALINE.

A new alkaloid has been added by Messrs. T. and H. Smith, of Edinburgh, to the already long list of alkaloids known to occur in opium. The new body has been named Xanthaline owing to the intense yellow colour of the salts which it forms with mineral acids. It is a weak base, insoluble in water, sparingly soluble in boiling water, but very easily in chloroform. Analyses point to the formula $C_{37} H_{36} N_2 O_9$. On treatment with nascent hydrogen it is converted into Hydroxanthaline, $C_{37} H_{38} N_2 O_9$, but on the whole it is a very stable substance, and not readily attacked by oxidising or other agents. Owing to its insolubility in water and the instability of its salts, it has not been possible to satisfactorily determine its physiological action.

